# Plasticity of Paneth cells and their ability to regulate intestinal stem cells

**DOI:** 10.1186/s13287-020-01857-7

**Published:** 2020-08-12

**Authors:** Xianglin Mei, Ming Gu, Meiying Li

**Affiliations:** 1 Department of Pathology, The Second Hospital of Jilin University, 218 Ziqiang Street, Changchun, 130041 China; 2 Department of Emergency and Critical Care Medicine, The Second Hospital of Jilin University, 218 Ziqiang Street, Changchun, 130041 China; 3 The Key Laboratory of Pathobiology, Ministry of Education, Jilin University, 126 Xinmin Street, Changchun, 130021 China

**Keywords:** Paneth cells, Intestinal stem cells, Lgr5+CBCs, Intestinal epithelium regeneration

## Abstract

Paneth cells (PCs) are located at the bottom of small intestinal crypts and play an important role in maintaining the stability of the intestinal tract. Previous studies reported on how PCs shape the intestinal microbiota or the response to the immune system. Recent studies have determined that PCs play an important role in the regulation of the homeostasis of intestinal epithelial cells. PCs can regulate the function and homeostasis of intestinal stem cells through several mechanisms. On the one hand, under pathological conditions, PCs can be dedifferentiated into stem cells to promote the repair of intestinal tissues. On the other hand, PCs can regulate stem cell proliferation by secreting a variety of hormones (such as wnt3a) or metabolic intermediates. In addition, we summarise key signalling pathways that affect PC differentiation and mutual effect with intestinal stem cells. In this review, we introduce the diverse functions of PCs in the intestine.

## Introduction

**Fig. 1:**
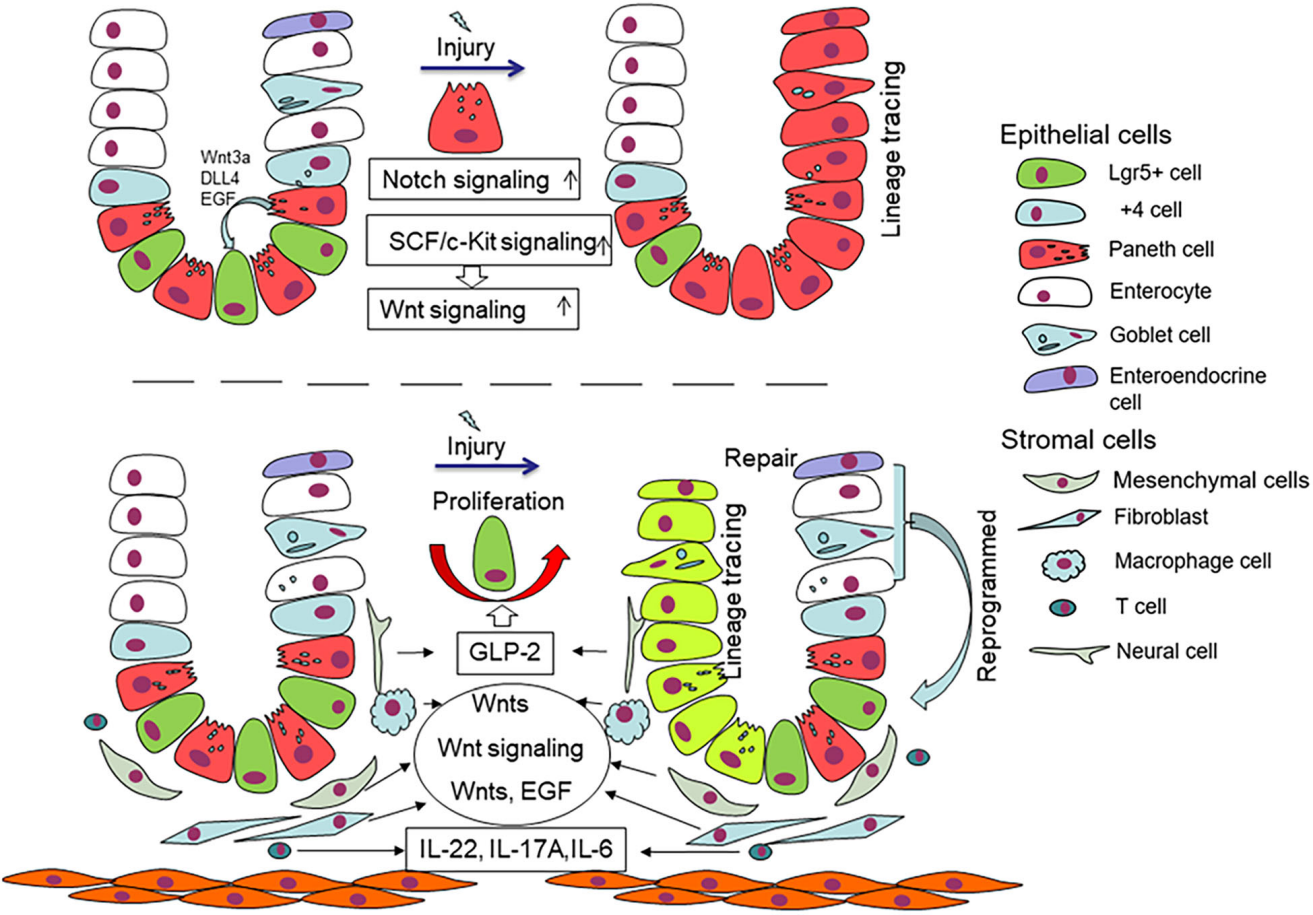
PCs located in crypt provide support to stem cells by secreting EGF, Wnt3a and DLL4. Under injury condition, PCs acquire stem features and generate all type of intestinal epithelial cells by activating Notch and Wnt signals. The lamina propria consists of multiple components and cell types, including mesenchymal cells, fibroblasts, neural cells, T cells and macrophage cells; they promote stem cell proliferation by activating Wnt signalling pathway or secreting cytokines (e.g. IL-22, IL-6 and GLP-2). Other differentiated cells such as enteroendocrine cell and enterocyte can restore stem features and promote damage repair

The intestinal epithelium, a single layer of columnar cells, lines the luminal surface of the intestinal mucosa and is regenerated throughout adult life. The intestinal epithelium is one of the most rapidly proliferating epithelia in mammals and plays an essential role in maintaining the balance between homeostasis and pathological condition [1]. In the small intestine, the epithelium comprises repeating crypt-villus units, 5–positive (Lgr5+) crypt base columnar (CBC) stem cells located in the crypt base with lysozyme-secreting Paneth cells (PCs) forming a mosaic pattern [2] (Fig. 1). The close interaction of CBCs with PCs is essential to maintain the stem cell function of CBCs.

In the normal physiological state, the Wnt signalling cascade is key pathway that regulates the proliferation and differentiation of intestinal stem cells (ISC) [2–5]. Lgr5 is a receptor for R-spondins and participates in canonical Wnt signalling [2]. Lgr5+CBCs undergo constant renewal to produce secretory cells and enterocytes to maintain the intestinal epithelial homeostasis and tissue integrity [6]. Although Lgr5+CBCs play an important role in daily intestinal maintenance, they are highly sensitive to injuries, such as inflammatory bowel disease (IBD) and irradiation [7]. A quiescent stem cell population residing at the +4 position functions as reserve stem cells upon depletion of the actively cycling stem cell pool (Lgr5+CBCs) [8].

Secretory lineages are derived from a common progenitor that emerges to occupy the +5-cell position above the stem cell niches [9]. A recent study corroborated that these short-lived precursors of PCs and enteroendocrine cells (intermediate cells) can dedifferentiate to Lgr5+ stem cells upon injury [10]. Further study demonstrated that fully committed PCs can dedifferentiate into a regenerative program to sustain intestinal epithelium homeostasis [1].

As part of the niche, PCs represent a population of morphologically distinct and functionally specialised intestinal epithelial cells [1], which have a lifespan of 3–6 weeks [11]. Wnt signalling, which is guided by EphB3 and partially antagonised by the Notch canonical pathway, induces the maturation of PCs and their migration downward into small intestinal crypts [12–14]. Matured PCs secrete microbicidal peptides, enzymes and growth factors [2]. The unique histo-morphological features and paracrine signalling implicate the special functions of PCs in cellular homeostasis as well as in the pathological state. In this paper, we provide an overview of recent studies that elucidate the important functions of PCs in intestinal epithelium regeneration, the role of PC interactions with intestinal stem cells and the key pathway that regulates PC development.

## Paneth cells mediate intestinal stem cell renewal and regeneration following homeostasis or injury

Intestinal stem cells are a source of rapid renewal of the intestinal epithelium by giving rise to all type intestinal epithelial lineages [6, 8]. Study on the relationship among intestinal stem cells, intestinal epithelial cells and stromal cells is mainly based on the application of animal models and organoid technology.

Irradiation animal models are a very widely used vector for intestinal inflammation damage. The mechanism involved in acute intestinal radiation is supported by evidence; that is, it mainly involves extensive activation of tumour necrosis factor alpha (TNFα) cascades, activation of tight junction signalling, aryl hydrocarbon receptor (AhR)-mediated apoptosis, activation of cell cycle signalling pathways and activation of the coagulation system [15, 16]. Dextran sulfate Na (DSS)-induced mouse enteritis model is also widely used and is similar to irradiation animal model that has high TNFα expression [17, 18]. These models provide a basis for studying intestinal homeostasis in animals and systematically determining the relationship between the intestinal epithelium and the body. Organoids of the small intestine are used to cultivate various types of intestinal epithelium in Petri dishes [19] and reveal the totipotent role of ISCs. This method can successfully separate the intestinal epithelium from other factors and can clearly elaborate the interaction between cells or intestinal epithelium and cytokines.

Intestinal stem cells are located in the intestinal crypts, where PCs are also located. Recent studies focused on defining the identity of intestinal stem cells and their interaction with their PC niche. Confocal cross-sectioning of the intestine crypt bottom showed an almost geometrical distribution of PCs and Lgr5 stem cells, leading to maximised heterotypic contact area (Paneth–stem cell) and minimised homotypic contact area [2]. This feature results in the dependence of CBCs on PC-mediated paracrine signalling.

In in vitro experimental research, PCs and Lgr5+ cells were sorted from untreated mice; their co-culture generated more organoids compared with Lgr5+ cells only [2]. In DSS-treated mouse model, coculture of PCs and Lgr5+ cells led to similar findings [20]. In vivo experiments partly reduce the number of PCs by mutation of Gfi1, transgenic expression of diphtheria toxin A and conditional deletion of Sox9; intestine stem cells were coincidently decreased in number and residual intestine stem cells colocalized with remaining PCs [2]. This finding demonstrates that Lgr5 stem cells compete for available PC surface; as such, PCs can promote the function of Lgr5+ cells.

In recent years, many studies have confirmed that PCs can regulate ISCs through various signalling pathways and growth factors. PCs itself could provide multiple secreted protein such as Wnt3, EGF and Notch ligand Dll4 and Dll1, which are crucial for stem-cell support [2, 11, 21] (Fig. 1). Wnt3 produced by PCs is amplified by the ubiquitous presence of R-spondin 1, which can be received by neighbour Lgr5 stem cells and promote the generation of organoids and the formation of asymmetry of crypt–villus [2]. A study proved that Wnt3 produced by PCs has spatial specificity for the role of ISC; Wnt3 did not freely diffuse but had direct contact with the Frizzled receptors to improve the function of Lgr5 stem cells [22]. Based on Wnt3 special secretion method, the division and distribution of PCs directly affect the function of ISC by activating the Wnt signalling pathway. Epidermal growth factor (EGF) is associated with intestinal proliferation and is also necessary for creating intestinal organoids [21]. Lrig1, as negative feedback regulator of the EGF receptor, is expressed with the highest levels in ISCs but is absent from PCs, when deletion in ISCs could lead to EGF-induced stem cell proliferation [23]. The EGF receptor (EGFR) is widely expressed in ISCs, while PCs could secrete its ligands with location specificity; this finding explains the regulatory effect of PCs on ISCs via the supply EGF ligand. Notch signalling is mediated through direct cell-to-cell contact of membrane-bound Notch ligands on one cell and transmembrane Notch receptors on adjacent cells; ISCs will lose their proliferation ability when Notch signalling is blocked [24]. DLL4 is a Notch ligand that is essential for the homeostasis of stem and progenitor cells and the simultaneous inactivation of Dll1 and Dll4, resulting in the complete conversion of proliferating progenitors into postmitotic goblet cells, concomitant with loss of stem cells (SCs) (Olfm4(+), Lgr5(+) and Ascl2(+)) [21]. Another study showed that Dll1 and Dll4 could be regulated by Fringe proteins, such as Lfng and Rfng; the latter is enriched in Paneth cells, and the former is mainly expressed in the villus; they promote cell surface expression of DLL1 and DLL4, further contributing to Lgr5+ ISCs self-renewal [25]. The influence of PCs on intestinal stem cells is similar to that of in vitro organoids. In organoid culture, Wnt, EGF and Noggin (antagonist of BMP) growth factors need to be added [21], while PCs can secrete Wnt and EGF, which are crucial for ISCs. These findings demonstrate that PCs are derived from paracrine signalling supporting intestinal stem niche.

However, another study reported a transgenic mouse model (Atoh1−/−; Atoh1 is a critical transcription factor for differentiation of PCs), which made the PCs completely removed possible. Three months after tamoxifen administration, Lgr5+ cells manifest intact long-term self-renewal in the gut epithelium in the absence of PCs. The levels of Wnt target transcripts, including genes associated with cell proliferation, such as CD44, Myc and Ccnd1, were unaffected in Lgr5+ stem cells. However, the Ki67 labelling index and the BrdU expression increased in Atoh1−/− mice [20]; this phenomenon may be caused by complete loss of PCs. In this regard, we should consider the other mechanism. In addition to PCs, subepithelial stromal Wnt signals (expressing Wnt2b, Wnt4 and Wnt5a) and other signals can maintain the epithelial stem cell pool in the absence of PCs under normal tissue homeostasis [26–30]. Besides, enteroendocrine and Tuft cells support Lgr5 stem cells by secreting Dll1 as an alternate source of Notch signal support during PC depletion [31].

## Contribution of Paneth cells to intestinal epithelium renewal

PCs that are terminally differentiated exit the cell cycle and do not express CBCs or proliferative markers during normal homeostasis in vivo [32, 33]. This phenomenon is also confirmed by mature PCs, which lack the capacity to form organoids if seeded individually [8]. Lysozyme (LYZ) is a characteristic marker of PCs in addition to CD24 and MMP7 [1]. Cell lineage tracing and RAN-seq analysis results demonstrate that PCs under pathological conditions (intestinal epithelium injury) may decrease or even lose the expression of these genes and obtain stem cell characteristics through de-differentiation, consequently contributing to intestinal regeneration [1, 13, 14] (Fig. 1). In irradiation mouse model, PCs can re-enter the proliferative state and then differentiate into all cell types of the intestinal epithelium. Gene set enrichment analysis (GSEA) showed that irradiated CD24+ PCs had a significantly stronger stem cell gene signature (e.g. Axin2, Lgr5, Ascl2, Olfm4) compared with non-irradiated counterparts [15]. The regenerative capacity of PCs is also maintained ex vivo; these cells can form small intestinal organoids when isolated from DSS-treated mice in the absence of Lgr5+ CBC cells [34]. Based on lineage tracing of doxorubicin (DXR)-treated mice, regenerative MUC2-positive goblet cells, chromogranin A-positive enteroendocrine cells and villin-positive enterocyte cells are all derived from PCs. These regenerative cells can no longer express PC markers, such as MMP7 and LYZ, indicating the total loss of the identity of PCs [35]. These demonstrated that PCs can acquire stem characteristics when the intestinal epithelium is injured, thereby achieving regeneration ability.

Notch and Wnt pathways play an important role in the plasticity of PCs. In animal and in vitro experiments, the Notch signalling pathway plays a key role in obtaining the stem characteristics of PCs. In vitro experiments, radiated PCs acquire stem cells features; this process leads to elevated expression of Notch pathway gene signatures, such as Hes1, Dtx4 and Adam17 compared with that under homeostatic conditions [35, 36]. Another study demonstrated that force activity Notch pathway in PCs can lead to dedifferentiation state [16]. In ex vivo study, the plasticity of PCs can be increased, under the Notch pathway was actively or passively activated condition. In vivo studies, Notch target genes, such as Notch intracellular domain (NICD) and Hes1, were activated in the small intestinal PCs of irradiated mice [33]. Forced NICD expression in PCs leads to transient proliferation states, followed by differentiation into other types of intestinal epithelial cells [33, 35]. Disintegrin and metalloproteinase domain-containing protein 10 (ADAM10) are necessary for lgr5+ CBC constitution when deletion can lead to crypt loss [26]. It is an a-secretase that activates the Notch pathway and is also important for PC plasticity. In DXR injury model, loss of ADAM10 blocked the ability of PCs to acquire the features of stem cells [37]. Hence, Notch signalling is necessary for PC dedifferentiation under injury conditions, such as radiation and chemical effects.

In the normal state, β-catenin is always nuclear positive in small intestinal PCs. β-catenin is a canonical target gene of the WNT signalling pathway, and it enters the nucleus and activates WNT signal. The positive nuclear expression of β-catenin in PCs probably indicates that PCs are sensitive to changes in Wnt signal within the crypt compartment. Acute loss of Wnt signalling results in depletion of PCs, whereas broad activation of canonical Wnt signalling results in the increase in the number of PCs [38, 39]. However, whether the Wnt/β-catenin pathway can affect the fate conversion of PCs remains controversial. Under homeostasis condition, forced expression of β-catenin in PCs cannot lead to acquiring stem cell-like features or change the life process of PCs [35]. The Wnt signal cannot affect the plasticity of PCs under steady-state conditions but participates in PC regeneration under pathological conditions. SCF, a ligand of the c-Kit receptor, is enhanced in patients with IBD and DSS (dextran sodium sulfate)-treated mouse model [40]. In DSS-treated mice, SCF/c-Kit signalling axis of the downstream PI3K/Akt through GSK3b inhibitory phosphorylation at Ser-9 increases the Wnt signalling activity in PCs [34]. Using SCF/c-Kit signalling axis inhibition agent can significantly reduce the number of organoids obtained from PCs alone; the same result was obtained using selective GSK3b inhibitor [34]. In this research, regardless of the method used, the proliferation and differentiation of PCs are achieved by activating the Wnt signalling pathway. By contrast, a homeostasis research indicated that adjusting no canonical Wnt signalling pathway can affect the ability of PCs to acquire stem-like features under pathological conditions. Hence, under injury condition, PCs can dedifferentiate and promote the regeneration of intestinal epithelium. This process needs Notch signal pathway activation and partly Wnt signal activation.

In addition to PCs, other differentiated intestinal epithelial cells, such as enterocyte cells, enteroendocrine cells (EECs) and other types of cells, can obtain stem features under certain conditions and promote intestinal injury repair. Enterocytes could acquire regeneration-associated genes by dedifferentiating into stem cells and Paneth-like cells when Lgr5+ stem cells are depleted [41]. A subset of neuroD1-derived EECs can reserve stem cell properties in response to radiation-induced injury or under homeostatic conditions [42]. Upon intestinal injury, Dll1- and Dll4-expressing cells generate absorptive and secretory cells [43].

## Stromal cells, inflammatory cells and cytokine mediators involved in intestinal regeneration

The lamina propria located below the intestinal epithelium of the intestine contains abundant mesenchymal cells, macrophages, immune cells and collagen. This area also plays an important role in repair of intestinal damage.

Stromal cells surround the base of crypts and mainly include mesenchymal cells, fibroblasts, endothelial cells, neural cells and inflammatory cells scattered in between them; stromal cells have an important regulatory effect on intestinal epithelium. Mesenchymal cells and related collagen are necessary for regeneration of the intestinal epithelium. The matrix-mediated integrin signalling via YAP/TAZ mechano-transduction promotes intestine regeneration in collagen type I; this process is accompanied by Wnt activation, and the same phenomenon was observed in human ulcerative colitis [44]. ISLR has been identified as a marker of mesenchymal stromal cells [45] because it can downregulate Hippo signals for YAP activation to promote intestine regeneration [46]. Mesenchymal stem cells (MSCs) have a significant therapeutic potential for tissue damage by improving the growth of intestinal crypts in vitro by activating the Wnt/β-catenin signalling pathway [47]. Similarly, stromal myofibroblasts through secreted Rspo3 proteins can stabilise the effects of Wnt ligands and further reprogram differentiated cells to support crypt regeneration upon damage [48]. A recent study showed that fibroblasts could secrete extracellular vesicles with Wnt and EGF activity, thereby rescuing Wnt- or EGF-deficient organoid growth [49]. Endothelial cells when inducing apoptosis did not affect intestine epithelial cell number change [50]. Glucagon-like peptide 2 (GLP-2) receptor, a product of neurons, could positively regulate epithelial growth [51]. We found that the regeneration of the intestinal epithelium induced by stromal cells is mainly achieved by directly or indirectly activating the Wnt signalling pathway.

Inflammatory cells such as T cells and macrophages, by produced cytokines, influence intestine regeneration. CD4+ and CD8+ T cells suppress human colon organoid formation; one of the mechanisms is T cells derived from IFNγ through JAK1/STAT1 activation directly target ISCs and induce apoptosis [52]. IFNγ also causes damages to PCs [53]. Colony-stimulating factor (CSF1) controls macrophage differentiation, and blockade of the CSF1 receptor-dependent macrophage leads to the conversion of Lgr5+ cells to reserve Bmi+ express ISC pool [54]. In addition, macrophages are one of the sources of Wnts in cases of intestinal radiation injury; macrophages derived from extracellular vesicles can transport Wnts to activate the Wnt pathway and promote intestinal epithelium repair and regeneration [55].

Cytokines, such as IL-4, IL-22, IL-6, IL-33 and tumour necrosis factor (TNF), are also involved in maintaining the function of the intestinal epithelium. IL-4 is a cytokine that is involved in T cell differentiation and regulation of immunoglobulin production [56]. In vitro experiments found that IL-4 can inhibit the development of organoids, as manifested by the decreased proliferation of ISCs and a significant reduction in PCs [57]; the mechanism is unclear, but the authors suggest that the dysfunction of PCs may be the cause of the reduction of ISCs. Th17 cells are a type of CD4+ T cells, which produce IL-22 or IL-17A and IL-6. These cytokines are important for regulating intestinal homeostasis upon inflammation [58, 59]. The IL-22 receptor (IL-22R) is present in intestinal epithelial cells (IEC), and IL-22 could augment ISC regeneration and reduction of allogeneic T cells caused intestine injury by positive regulation of STAT3; this process is independent of Wnt signalling pathway, Notch pathway and Paneth cells’ regulation [60]. Furthermore, IL-22 upregulates ATF3 (a stress-response molecule that exists in epithelial cells) by negatively targeting the protein tyrosine phosphatases (PTPs) of STAT3 to promote intestinal cell proliferation when knockout of ATF3 lead to reduction in the numbers of PCs and their granules [61]. IL-6 could be produced by epithelial cells and is a strong STAT3 inducer similar to IL-22; IL-6 is also positively regulated by ATF3 [59]. IL-6 signalling could also modulate crypt homeostasis, and the IL-6 receptor is only expressed in PCs by exotic and autocrine IL-6 specific induce activation of pSTAT3, via the Wnt signalling pathway, thereby increasing the proliferation of ISCs [62]. IL-33, a product of pericryptal fibroblasts, induces PC expansion via the ST2 receptor and indirectly stimulates intestinal stem cell proliferation; the Notch signalling pathway is involved in this process [63]. TNF is a central regulator of inflammation and is derived from bone marrow by interacting with TNF receptors located in the intestinal epithelium and induces the Wnt/β-catenin target gene expression for maintenance of intestinal ISCs [64]. The role of cytokines and immune cells on intestinal epithelium is also mainly reflected on the activation of the Wnt signalling pathway, and some of these cytokines are related to PC function and number change.

The regulation of intestinal epithelium is a comprehensive and complex process. In addition to the intestinal epithelium, sub-epithelium tissues and cytokines also provide support for the steady state of the intestinal epithelium. The activation of the Wnt pathway is necessary for ISCs to promote intestinal damage repair (Fig. 1).

## Role of Paneth cells in metabolic-mediated stem cell function

Cell metabolism has been implicated in stem cell maintenance and differentiation in adult stem cell populations [65]. Recent studies show that nutrients play an important role in maintaining the function of ISCs. For example, enhanced cholesterol biosynthesis leads to the proliferation of ISCs [66]. Low levels of vitamin D compromise the function of ISCs [67]. Fatty acid oxidation (FAO) enhances the function of ISCs [68]. Ketone body beta-hydroxybutyrate (βOHB) through class I HDAC inhibition activates the Notch pathway to promote the function of ISCs [69]. Glutamine promotes cell proliferation and mRNA expression of Lgr5+ cells [70].

The mechanistic target of rapamycin (mTOR) is an important nutrition sensing element. It could regulate intestinal epithelial cells and ISCs during injury conditions [71, 72]. These processes are canonical WNT pathway and Notch pathway independent [7]. mTOR could regulate intestinal epithelial cells and ISCs under injury conditions [71, 72]. mTOR complex 1 (mTORC1) is an important regulator for PCs and goblet cell differentiation; increase in mTORC1 can lead to a marked decrease in both type of cells, while decrease in mTORC1 will lead to PCs smaller in size and intracellular secretory granules [73, 74]. Recent studies have further confirmed that mTORC1 is involved in the caloric metabolic process of ISCs and PCs [75].

**Fig. 2:**
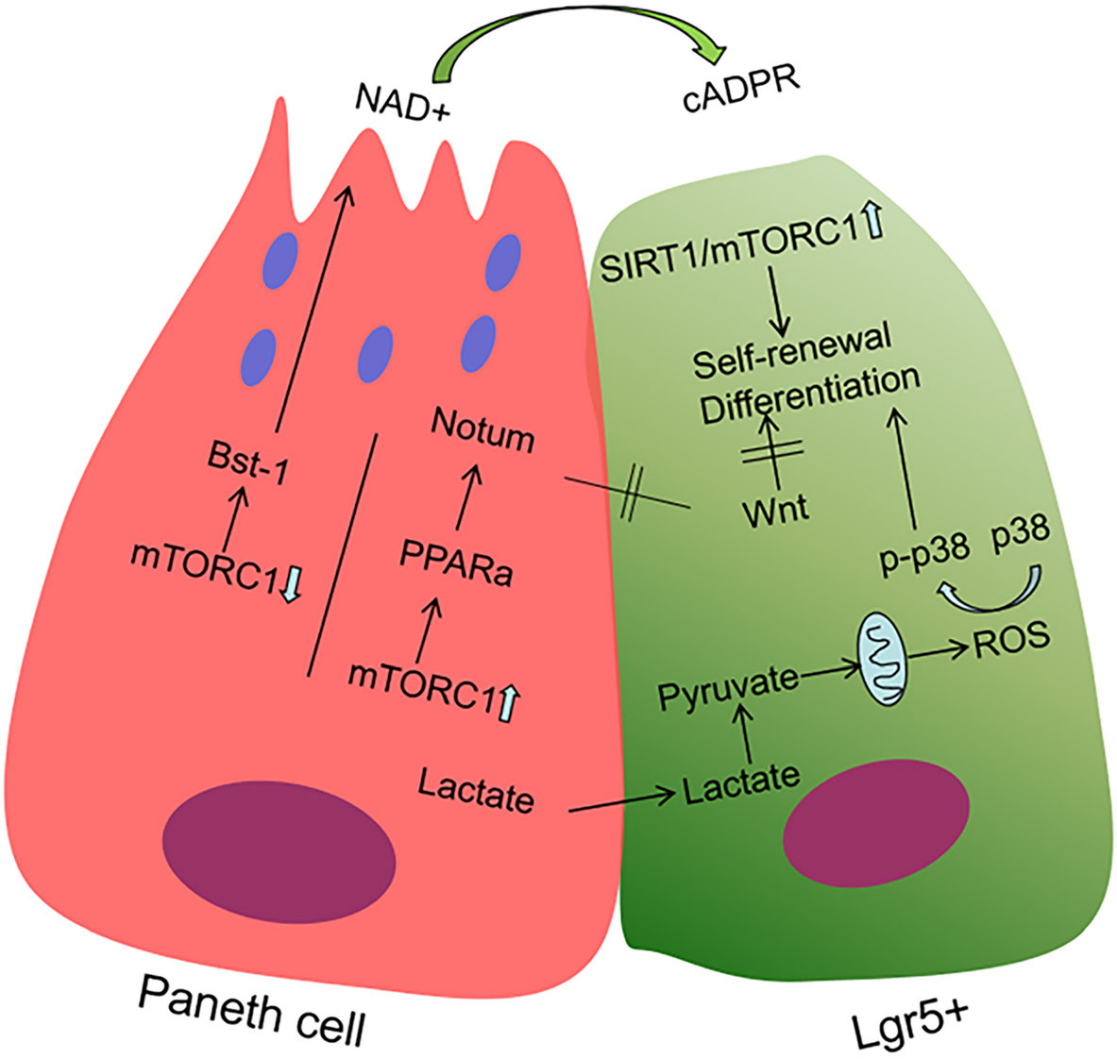
Schematic of the model for PC-mediated stem cell function. PCs augment stem cell function response to calorie restriction by reducing mTOR and supplying respiratory substrates as by-product of lactate. It can also suppress ISC function by increasing mTOR in ageing

PCs can affect the proliferation and differentiation of ISCs through different metabolic pathways. Under conditions of caloric restriction in mice, the numbers of PCs and Lgr5+ CBCs increased in vivo. In ex vivo study, co-culture with PCs from caloric restriction mice with intestine stem cell (ISCs from untreated or treated mice) gave rise to more and larger secondary organoid bodies than used normal PCs. The mechanism through which PCs augment stem cell function in response to calorie restriction involves the reduction of mTORC1 signalling in the cytoplasm. Under caloric restriction, downregulation of mTORC1 enhances the release of the paracrine effector bone stromal antigen 1 (Bst-1), which converts NAD+ to cyclic ADP ribose (cADPR) and promotes CBC proliferation [76] (Fig. 2). Further research has shown that PCs can increase NAD synthesis and then enhance SIRT1 (a NAD-dependent protein deacetylase) and mTORC1 activity to promote ISC proliferation and protein synthesis (Fig. 2) [75]. Based on gene expression profiling, no changes were observed in pathways, such as Wnt or Notch, that were previously implicated in mediating the interaction between PCs and ISCs [76]. In situ glycan editing method found that high LacNAc (a sugar structure, which is linked to the cell surface protein PSGL-1 (P-selectin glycoprotein ligand) [77]) expression on the surface of Paneth cells can block LacNAc, causing ISC proliferation arrest and accompanied by Wnt and Notch target gene changes [78].

In other studies, PCs affect the function of stem cells through other metabolic pathways. Study elaborated that Lgr5+ CBCs and PCs from the small intestine of mice displayed different metabolic programs. Compare with PCs, Lgr5+ CBCs showed higher mitochondrial activity. In Lgr5+ CBCs, the onset of differentiation relies on mitochondrial oxidative phosphorylation (OXPHOS), through which the number of crypts per organoid and the number of PCs increase. Treatment with glycolysis inhibitors in PCs strongly reduce their niche-supporting function and organoid formation. Addition of exogenous lactate, an end product of glycolysis, can strongly enhance the ability of Lgr5+ CBCs to form organoids [79]. This experiment confirmed that PCs could provide the respiratory substrate by converting lactate into pyruvate to sustain OXPHOS in Lgr5+ CBCs, leading to ROS signalling, activation of p38 and cell differentiation [79] (Fig. 2).

In conclusion, Paneth cells serve as sensors for nutritional status affects the functions of Lgr5+ cells not only through growth factor signalling but also by providing them with a metabolic fuel source.

## Paneth cells affect intestinal stem cells in ageing

Ageing is an inevitable biological event and is usually accompanied by decreased function, including diminished self-renewal ability of stem cells [80]. This process involves various systems of the human body (e.g., haematopoietic system, skin, brain) and may have clinical consequences, such as defective immune responses, Alzheimer’s disease and hair loss [81–83]. The molecular mechanism of ageing remains unclear. A large number of recent studies believe that chromosome instability, pro-inflammation and mTOR play an important role in the occurrence of ageing and related diseases [84, 85]. In the intestinal epithelium, the features of ageing are accompanied by cell cycle changes, oxidative stress and enhanced apoptosis [86]. Ageing of ISCs is driven by mTORC1 [87] as well as decreased activity of the Wnt signalling pathway [88]. In the gut, ageing impairing the balance between stem cell reserve and differentiation [80, 89]. In study of mouse models, the small intestine of ageing mice showed a decrease in crypt number accompanied by an increase in crypt length and width compared with those in young mice [88]. Increasing the number of terminally differentiated cells, such as PCs and goblet cells, also alters the differentiation ability of stem cells [88]. Flow cytometry analysis further confirmed that the number of Lgr5+ cells in the intestinal crypts of ageing mice was significantly reduced, whereas the number of PCs was significantly increased [90]. Whether PCs are involved in ageing and their effect on crypt stem cells can be validated by co-culture. Crypts cultured from old mice yielded fewer and less complex organoids than those from young mice [90]. Co-culture of Lgr5+ cells from old mice with PCs from young mice led to higher organoid-forming capacity than culture with PCs from old mice [90]. The same result was obtained in long-term co-culture. These phenomena indicate that PCs can affect intestinal ageing [90]. During physiological ageing, canonical Wnt signalling declined in ISCs [88]. In a similar study on old PCs, RNA sequencing showed specific deregulation of genes that encode secreted or plasma-membrane-associated proteins; however, Wnt-responsive genes and the expression of Wnt3 or EGF were not significantly altered in old PCs [38]. This phenomenon shows that the impact of PCs on ISCs is not achieved through the WNT signalling pathway. Another study showed that downregulated genes in aged ISCs included, as anticipated, cell proliferation but also extracellular matrix, PPAR, SMAD signalling and Wnt signalling pathways [74]. The extracellular Wnt inhibitor Notum was significantly upregulated in PCs from ageing mice [88, 91]. Notum is regulated by the canonical Wnt pathway, forming a negative-feedback loop that was significantly upregulated in old PCs [92]. The mTORC1 expression decreased under caloric restriction condition, leading to the proliferation of CBCs. This signalling is also linked with ageing with an increase in mTORC1 [74]. In ageing, activation of the downstream of mTORC1 inhibited PPAR-α and increased the Notum expression in PCs of the ISC niche in mouse and human, thereby inhibiting Wnt signalling and reducing stem cell maintenance and regeneration. Reversing observed changes in mTORC1–PPAR-α signalling restored epithelial regeneration [74] (Fig. 2). PCs can metabolically mediate stem cell function. Metabolic changes have an unexpected effect on ageing; fasting and caloric restriction could improve the function of ISCs with or without PCs involved. Fasting can enhance ageing intestinal stem cell function by inducing fatty acid oxidation (FAO)-PPAR axis without PCs [2]. While the activation of the NAD/SIRT1/mTORC1 axis promotes ISC function under conditions of caloric restriction, the function of ageing ISCs can be restored using NAD analogues; this process needs PCs [93].

These data demonstrate that Notum produced by PCs attenuates the regenerative capacity of ageing intestinal epithelium in vivo by reducing Wnt activity in stem cells. PCs in contrast can promote the function recovery of ageing ISCs when fasting by metabolic-related pathways.

## Growth factor-mediated signalling pathways regulate the development or function of Paneth cells

Alterations in the number and function of PCs are associated with maintaining intestinal homeostasis. Thus, any mechanism that affects PC development or function could further change the intestinal homeostasis. In mice, PCs in the intestine differentiate around postnatal day 14 [94]. In humans, PCs differentiate around week 20 of foetal gestation [95, 96]. Wnt and Notch are two of the main signalling pathways that control the differentiation of ISCs; the differentiation of PCs is initially controlled through a Notch-dependent mechanism during secretory progenitor specification; further PC maturation is regulated by Wnt signalling [26, 97]. PCs derived from a subset of Lgr5+ label-retaining cells (LRCs) were proposed to constitute secretory precursors for PCs and goblet cells [8, 9] (Fig. 1).

**Fig. 3:**
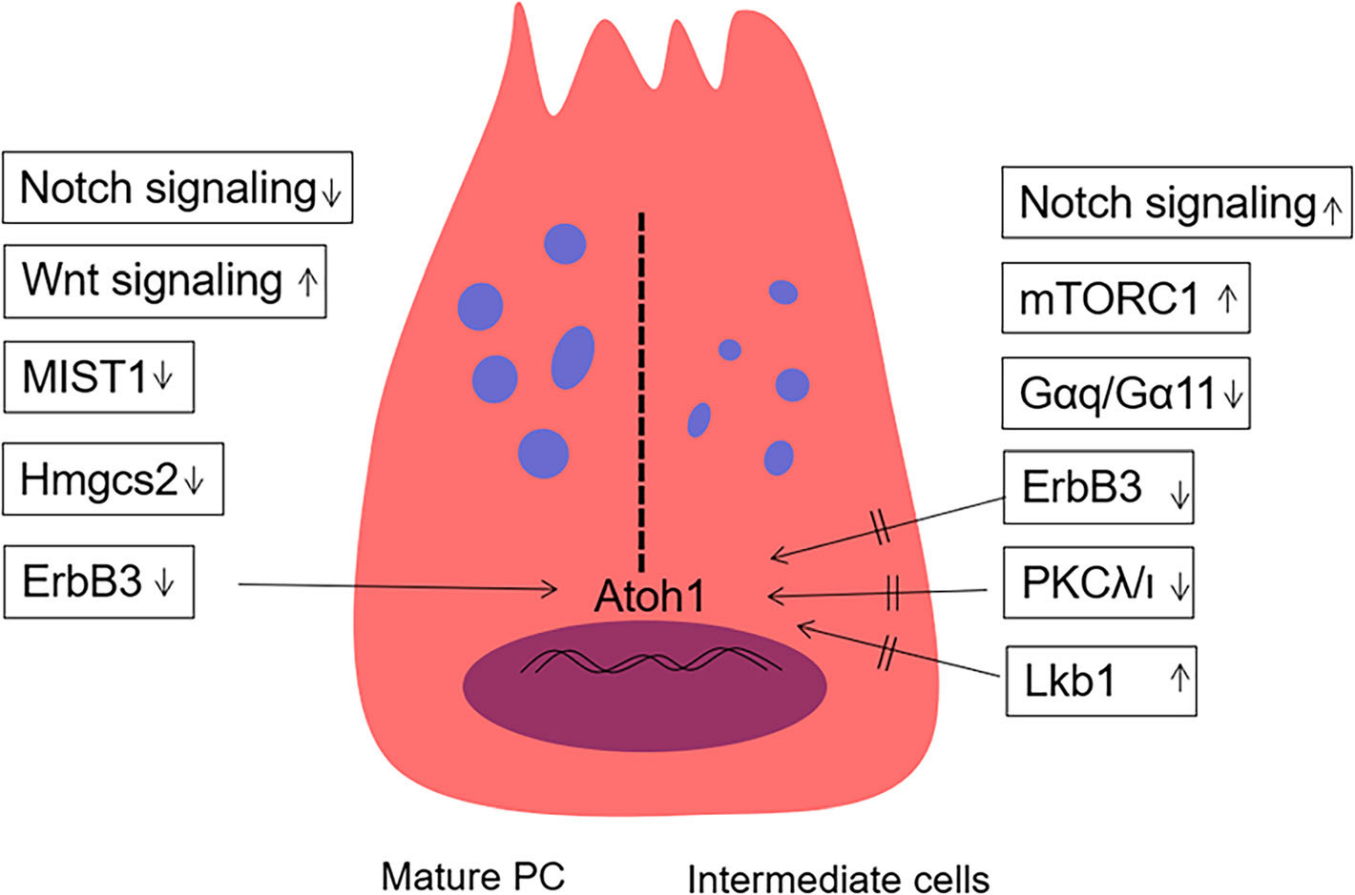
Schematic of the model for PC regulation. Wnt signalling upregulates and downregulates Notch signalling, ErbB3, MIST1and Hmgcs2 can promote PC maturation. Notch signalling, mTORCC1 and Lkb1 upregulated or ErbB3, Gαq/Gα11 and PKCλ/ι inactivation inhibit PCs differentiation and function

The Wnt signalling pathway is vital to promote PC development and is also important for maintaining the undifferentiated state of intestinal crypt progenitor cells. Administration of Wnt3a increases the number of PCs in intestinal organoids [26] (Fig. 3). In embryonic intestine, the development of PCs requires the activation of the Wnt/β-catenin signalling pathway [12]. In the adult small intestine, the selective switch off/on of the Wnt/β-catenin signalling pathway in LRCs induced the transition of PCs to goblet cells [98]. The mechanism involved is that Shp2 mediates MAPK signalling, thereby controlling the regulation of Wnt/β-catenin activity to decide the final fates of goblet cells and PCs [99]. In detail, high Shp2/MAPK activity decreased the Wnt/β-catenin signals, promoting goblet cell differentiation; meanwhile, Shp2 ablation increased Wnt/β-catenin activity, promoting PC differentiation [99]. In addition to PCs, which can secrete Wnt3, other intestinal cells, such as stromal cells, can secrete a variety of Wnt proteins [36, 37]. The secreted Wnt proteins are transported by the multi-pass transmembrane G protein-coupled receptor 177 (Gpr177) and partly dependent on Rab8a-mediated anterograde transport for exocytosis [100, 101]. Deletion of Rab8a significantly reduced the number of LYZ+ PCs, and this phenomenon may block the terminal differentiation of PCs from the precursors [102]. Hence, deletion of Rab8a and Shp2/MAPK can affect the Wnt pathway and determine the maturation of PCs.

Besides the Wnt signalling pathway, many other growth factors affect the development of PCs]. The ErbB3 neuregulin (NRG) receptor is expressed in most epithelial tissues [103]. In intestinal epithelial cells with knocked out ErbB3, PCs are generated in advance as early as postnatal day 7, while wild-type mouse PCs appear at around 2 weeks after birth [94, 104]. The number of mature PCs and MMP7/MUC2+ intermediate cells increased in adult mouse ileum [104]. The underlying mechanism is that ErbB3 regulates PC differentiation through the PI3K–Akt pathway. The PI3K-mediated suppression of Atoh1 (which is required for secretory fate determination) inhibits PC differentiation [104]. Another growth factor named helix-loop-helix transcription factor (MIST1) is a scaling factor; this factor can control the fate of exocrine cells (such as pancreatic acinar cells, zymogenic cells of the stomach) to affect secretory capacity [105]. In the small intestine, the absence of MIST1 increased the numbers of PCs, which exhibit an LYZ/MUC2+ intermediate cell phenotype and morphologically show immature features, including decreased granule size and distended rough endoplasmic reticulum [105, 106] (Fig. 3). The Gαq/Gα11 signalling exerts its actions on G-protein-coupled receptors; the absence of Gαq/Gα11 signalling impairs the maturation of Paneth cells (enlarged, increased in number) and reduces Wnt signalling [107].

The Notch signalling pathway plays a significant role in controlling the cell fate of intestinal epithelial progenitor cells into absorptive and secretory lineages. The inhibition of the Notch pathway by either chemical inhibition or knockout of associated genes (e.g. Notch1, Notch2, Dll1 and Dll4) can increase the secretory phenotype of cells, mainly goblet cells [97, 108–110]. The downregulation of the Notch signalling pathway promotes secretory precursors differentiating into goblet cells but may only slightly or indirectly affect the differentiation of PCs. Progenitor cells can differentiate into PCs through downregulation of the Notch signalling pathway [111]. DAPT, a selective γ-secretase inhibitor of the Notch pathway, induces the expression of LYZ and Defa6 (another marker of PCs differentiation) when incubated with intestinal organoids [112]. This effect on the differentiation of PCs may be achieved by PKCλ/ι (a member of atypical PKC family, negative regulation PCs differentiation) by changing the epigenetic genetics of Atoh1 upon Notch inactivation [112] (Fig. 3). A recent study showed that acute Notch inhibition leads to rapid apoptosis of PCs, while Notch activation counteracts the death of PCs caused by caspase-8 (casp8) absence; these finding suggests that Notch signalling are required for PC maintenance [43, 113].

In addition to above signalling pathways, metabolism-related growth factors determined regulate the fate of PCs. Ketone bodies can mediate the proliferation ability of ISCs; 3-hydroxy-3-methylglutaryl-CoA synthase 2 (Hmgcs2) is the gene encoding the rate-limiting step for ketogenesis when the deletion of Hmgcs2 leads to pronounced expansion of PCs [9, 114]. Tumour suppressor and kinase Lkb1 (encoded by Stk11) is a bioenergetic sensor that controls cell metabolism through repression of the transcription of Atoh1, thereby restricting the differentiation of ISCs into secretory lineage [115]. mTORC1, as a nutrient sensor, also controls PC differentiation in the intestinal epithelium; the inactivation of mTORC1 will reduce the function and number of PCs. Prohibitin 1 (PHB1), a mitochondrial membrane component protein, is crucial for maintaining PCs; when loss will lead to defect of PCs, other factors, such as inflammation-associated mitochondrial dysfunction, also produce the same effect [116, 117].

Inflammatory factors are also involved in the regulation of PCs. For example, the deletion of Toll-like pattern recognition receptor (TLRs)9 could lead to downregulation of the number of PCs by blocking the release of interleukin17A (IL-17A) [118]. The IL-22/Stat3 pathway can improve PC function by increasing the protein levels of lysozyme, RegIIIγ and α-cryptdin 5 under parenteral nutrition [119]. The IL-4 treatment can significantly decrease the gene expression levels of Paneth cell marker LYZ and reduce the proliferation of ISCs [120]. In addition, the IκB kinase (IKK)-NF-κB signalling pathway is involved in inflammatory process when its activation caused the depletion of PCs [121]. Furthermore, STAT5-dependent JAK2 signalling and JAK1-dependent STAT1 signalling are required for anti-inflammatory cytokine; the absence of STAT5 or the activation of STAT1 decreases the number of PCs [57, 122]. Indoleamine 2,3-dioxygenase 1 (IDO1) was upregulated by inflammatory cytokines, thereby promoting the proliferation of PCs; this process blocks the activation of Notch1 [123].

We have discussed many growth factors and inflammatory factors that can mediate the development of PCs in different angles. The occurrence and differentiation of PCs is greatly affected by the Wnt signalling pathway but is minimally influenced by the Notch signalling pathway. In addition to traditional pathways, metabolic and inflammatory processes also affect the fate of PCs to a certain extent.

## Conclusion

PCs are a group of specialised epithelial cells of the small intestine and contain multiple secretory granules filled with antimicrobial peptides, which are essential for control of microbial growth and maintaining intestinal integrity. Previous studies and reviews focused on how PCs shape the intestinal microbiota or response to the immune system [124–126]. This paper provides an overview of the function of PCs and their contribution to ISC maintenance during intestinal homeostasis and injury condition.

PCs are located at the bottom of intestinal crypts. As a terminally differentiated cell, the function of ISCs are partially regulated by paracrine-specific secreted proteins (Wnt, EGF) or metabolic regulation under the conditions of intestinal homeostasis, including ageing and caloric restriction. The regulation of ISCs is mainly achieved by regulating the Wnt signalling pathway, but the metabolic regulation process, which promotes stem cell function by providing metabolic substrates. In the pathological state, the function of ISCs is enhanced, and PCs can acquire stem features to repair the intestinal mucosal epithelium. The strong plasticity of PCs is mainly achieved by reactivating the Notch signalling pathway.

In the whole intestine, various cells of the intestinal lamina propria and cytokines play an important role in the steady state of ISCs and the regulation of PCs. As mesenchymal cells, fibroblasts can promote the function of ISCs by activating the Wnt signalling pathway or secreting Wnt and EGF; this process can partially overlap with the function of PCs. Similar to stromal cells, cytokines (e.g. IL-6, IL-33 and TNF) also promote the proliferation of ISCs by activating Wnt.

The development of PCs is also regulated by a variety of growth factors and cytokines especially under pathological conditions; the differentiation of precursor stem cells into PCs is greatly affected. PCs play an important role in the function of ISCs, in the ways that regulate PCs, which also means a certain effect on ISCs. The main factor that effects the differentiation of PCs is the Wnt signalling pathway.

In summary, PCs can be regarded as the guardians of intestinal crypt function and have a huge regulatory effect on ISCs under pathological and physiological conditions. The interaction network between PCs and stromal cells and between PCs and differentiated intestinal epithelial cells remains unclear.

## Data Availability

Not applicable.

## Abbreviations

PCs: Paneth cells
CBC: Crypt base columnar
ISCs: Intestine stem cells
IBD: Inflammatory bowel disease
LYZ: Lysozyme
OXPOS: Mitochondrial oxidative phosphorylation
LRCs: Lgr5+ label-retaining cells
mTORC1: mTOR complex 1
MIST1: Helix-loop-helix transcription factor
Bst-1: Bone stromal antigen 1
cADPR: Cyclic ADP ribose
NICD: Notch intracellular domain
CSF1: Colony-stimulating factor
TNF: Tumour necrosis factor
FAO: Fatty acid oxidation

## References

[1] Clevers H (2013). The intestinal crypt, a prototype stem cell compartment. Cell.

[2] Basak, O., van de Born, M., Korving, J., Beumer, J., van der Elst, S., van Es, et al. Paneth cells constitute the niche for Lgr5 stem cells in intestinal crypts. Nature. 2011; 469, 415–418. 10.1038/nature09637 PMC3547360 21113151

[3] Clevers H, Nusse R (2012). Wnt/β-catenin signaling and disease. Cell.

[4] Clevers H, Loh KM, Nusse R. An integral program for tissue renewal and regeneration: Wnt signaling and stem cell control. Science. 2014;346(6205):1248012. 10.1126/science.1248012 25278615

[5] Karin M, Clevers H (2016). Reparative inflammation takes charge of tissue regeneration. Nature.

[6] Barker N, van Es JH, Kuipers J, Kujala P, van den Born M, Cozijnsen M, Haegebarth A, Korving J, Begthel H, Peters PJ, Clevers H (2007). Identification of stem cells in small intestine and colon by marker gene Lgr5. Nature.

[7] Goodell MA, Nguyen H, Shroyer N (2015). Somatic stem cell heterogeneity: diversity in the blood, skin and intestinal stem cell compartments. Nat Rev Mol Cell Biol.

[8] Takeda N, Jain R, LeBoeuf MR, Wang Q, Lu MM, Epstein JA (2011). Interconversion between intestinal stem cell populations in distinct niches. Science.

[9] van Es JH (2012). Dll1+ secretory progenitor cells revert to stem cells upon crypt damage. Nat Cell Biol.

[10] Buczacki SJA, Zecchini HI, Nicholson AM, Russell R, Vermeulen L, Kemp R, Winton DJ (2013). Intestinal label-retaining cells are secretory precursors expressing Lgr5. Nature.

[11] Ireland H, Houghton C, Howard L, Winton DJ (2005). Cellular inheritance of a Cre-activated reporter gene to determine Paneth cell longevity in the murine small intestine. Dev Dyn.

[12] van Es JH, Jay P, Gregorieff A, van Gijn ME, Jonkheer S, Hatzis P (2005). Wnt signaling induces maturation of Paneth cells in intestinal crypts. Nat Cell Biol.

[13] Batlle E, Henderson JT, Beghtel H, van den Born MM, Sancho E, Huls G, Meeldijk J (2002). Beta-catenin and TCF mediate cell positioning in the intestinal epithelium by controlling the expression of EphB/ephrinB. Cell..

[14] Demitrack ES, Samuelson LC (2016). Notch regulation of gastrointestinal stem cells. J Physiol.

[15] Zheng J, Wang J, Pouliot M, Authier S, Zhou D, Loose DS, Hauer-Jensen M (2015). Gene expression profiling in non-human primate jejunum, ileum and colon after total-body irradiation: a comparative study of segment-specific molecular and cellular responses. BMC Genomics.

[16] Liang W, Leibowitz BJ, Wang X, Epperly M, Greenberger J, Zhang L, Yu J (2016). Inhibition of CDK4/6 protects against radiation-induced intestinal injury in mice. J Clin Invest.

[17] Laroui H, Ingersoll SA, Liu HC, Baker MT, Ayyadurai S, Charania MA (2012). Dextran sodium sulfate (DSS) induces colitis in mice by forming nano-lipocomplexes with medium-chain-length fatty acids in the colon. PLoS One.

[18] Obermeier F, Kojouharoff G, Hans W, Schölmerich J, Gross V, Falk W (1999). Interferon-gamma (IFN-γ)- and tumour necrosis factor (TNF)-induced nitric oxide as toxic effector molecule in chronic dextran sulphate sodium (DSS)-induced colitis in mice. Clin Exp Immunol.

[19] Sato T, Vries RG, Snippert HJ (2009). Single Lgr5 stem cells build crypt-villus structures in vitro without a mesenchymal niche. Nature.

[20] Kim TH, Escudero S, Shivdasani RA (2012). Intact function of Lgr5 receptor-expressing intestinal stem cells in the absence of Paneth cells. Proc Natl Acad Sci U S A.

[21] Poulsen SS, Nexo E, Olsen PS, Hess J, Kirkegaard P (1986). Immunohistochemical localization of epidermal growth factor in rat and man. Histochemistry.

[22] Farin HF, Jordens I, Mosa MH, Basak O, Korving J, Tauriello DVF (2016). Visualization of a short-range Wnt gradient in the intestinal stem-cell niche. Nature.

[23] Wong VW, Stange DE, Page ME, Buczacki S, Wabik A, Itami S (2012). Lrig1 controls intestinal stem-cell homeostasis by negative regulation of ErbB signaling. Nat Cell Biol.

[24] VanDussen KL, Carulli AJ, Keeley TM, Patel SR, Puthoff BJ, Magness ST, Tran IT, Maillard I, Siebel C, Kolterud Å, Grosse AS, Gumucio DL, Ernst SA, Tsai YH, Dempsey PJ, Samuelson LC (2012). Notch signaling modulates proliferation and differentiation of intestinal crypt base columnar stem cells. Development..

[25] Murthy PKL, Srinivasan T, Bochter MS, Xi R, Varanko AK, Tung K-L (2018). Radical and lunatic fringes modulate notch ligands to support mammalian intestinal homeostasis. Elife.

[26] Farin HF, Van Es JH, Clevers H (2012). Redundant sources of Wnt regulate intestinal stem cells and promote formation of Paneth cells. Gastroenterology.

[27] Gregorieff A, Pinto D, Begthel H, Destree O, Kielman M, Clevers H (2005). Expression pattern of Wnt signaling components in the adult intestine. Gastroenterology.

[28] Hoshkes-Carmel M (2018). Subepithelial telocytes are an important source of Wnts that supports intestinal crypts. Nature.

[29] Degirmenci B, Valenta T, Dimitrieva S, Hausmann G, Basler K (2018). GLI1-expressing mesenchymal cells form the essential Wnt-secreting niche for colon stem cells. Nature.

[30] Miyoshi H, Ajima R, Luo CT, Yamaguchi TP, Stappenbeck TS (2012). Wnt5a potentiates TGF-beta signaling to promote colonic crypt regeneration after tissue injury. Science..

[31] van Es JH, Wiebrands K, López-Iglesias C, van de Wetering M, Zeinstra L, van den Born M (2019). Enteroendocrine and tuft cells support Lgr5 stem cells on Paneth cell depletion. Proc Natl Acad Sci U S A.

[32] JH, Clevers H (2014). Mapping early fate determination in Lgr5+ crypt stem cells using a novel Ki67-RFP allele. EMBO J.

[33] Jones JC, Brindley CD, Elder NH, Myers MG (2019). Cellular plasticity of Defa4Cre-expressing Paneth cells in response to notch activation and intestinal injury. Cell Mol Gastroenterol Hepatol.

[34] Schmitt M, Schewe M (2018). Paneth cells respond to inflammation and contribute to tissue regeneration by acquiring stem-like features through SCF/c-kit signaling. Cell Rep.

[35] Yu S, Tong K, Zhao Y (2018). Paneth cell multipotency induced by notch activation following injury. Cell Stem Cell.

[36] Sancho R, Cremona CA, Behrens A (2015). Stem cell and progenitor fate in the mammalian intestine: Notch and lateral inhibition in homeostasis and disease. EMBO Rep.

[37] Tsai YH, VanDussen KL, Sawey ET, Wade AW, Kasper C, Rakshit S, Bhatt RG, Stoeck A, Maillard I, Crawford HC, Samuelson LC, Dempsey PJ (2014). ADAM10 regulates Notch function in intestinal stem cells of mice. Gastroenterology.

[38] Pinto D, Gregorieff A, Begthel H, Clevers H (2003). Canonical Wnt signals are essential for homeostasis of the intestinal epithelium. Genes Dev.

[39] Andreu P, Peignon G, Slomianny C, Taketo MM, Colnot S, Robine S, Lamarque D, Laurent-Puig P, Perret C, Romagnolo B (2008). A genetic study of the role of the Wnt/beta-catenin signaling in Paneth cell differentiation. Dev Biol.

[40] Rothenberg ME, Nusse Y, Kalisky T, Lee JJ, Dalerba P, Scheeren F, Lobo N, Kulkarni S, Sim S, Qian D (2012). Identification of a cKit(+) colonic crypt base secretory cell that supports Lgr5(+) stem cells in mice. Gastroenterology.

[41] Tetteh PW, Basak O, Farin HF, Wiebrands K, Kretzschmar K, Begthel H (2016). Replacement of lost Lgr5-positive stem cells through plasticity of their enterocyte-lineage daughters. Cell Stem Cell.

[42] Sei Y, Feng J, Samsel L, White A, Zhao X, Yun S (2018). Mature enteroendocrine cells contribute to basal and pathological stem cell dynamics in the small intestine. Am J Physiol Gastrointest Liver Physiol.

[43] Bohin N, Keeley TM, Carulli AJ, Walker EM, Carlson EA, Gao J, et al. Rapid crypt cell remodeling regenerates the intestinal stem cell niche after Notch inhibition. Stem Cell Rep. 2020;14;15(1):156-170. 10.1016/j.stemcr.2020.05.010 PMC7363878 32531190

[44] Yui S, Azzolin L, Maimets M, Pedersen MT, Fordham RP, Hansen SL (2018). YAP/TAZ-dependent reprogramming of colonic epithelium links ECM remodeling to tissue regeneration. Cell Stem Cell.

[45] Maeda K, Enomoto A, Hara A, Asai N, Kobayashi T, Horinouchi A, Maruyama S, Ishikawa Y, Nishiyama T, Kiyoi H (2016). Identification of meflin as a potential marker for mesenchymal stromal cells. Sci Rep.

[46] Xu J, Yang T, Sheng X, Tian Y, Deng M, Sujuan D (2020). Secreted stromal protein ISLR promotes intestinal regeneration by suppressing epithelial Hippo signaling. EMBO J.

[47] Gong W, Guo M, Han Z, Wang Y, Yang P, Chang X (2016). Mesenchymal stem cells stimulate intestinal stem cells to repair radiation-induced intestinal injury. Cell Death Dis.

[48] Harnack C, Berger H, Antanaviciute A, Vidal R, Sauer S, Simmons A (2019). R-spondin 3 promotes stem cell recovery and epithelial regeneration in the colon. Nat Commun.

[49] Oszvald Á, Szvicsek Z, Sándor GO, Kelemen A, Soós AÁ, Pálóczi K (2020). Extracellular vesicles transmit epithelial growth factor activity in the intestinal stem cell niche. Stem Cells.

[50] Takashima S, Martin ML, Jansen SA, Fu Y, Bos J, Chandra D. T-cell-derived interferon-γ programs stem cell death in immunemediated intestinal damage. Sci Immunol. 2019;4(42):eaay8556. 10.1126/sciimmunol.aay8556 PMC7239329 31811055

[51] Paris F, Fuks Z, Kang A (2001). Endothelial apoptosis as the primary lesion initiating intestinal radiation damage in mice. Science..

[52] Bjerknes M, Cheng H (2001). Modulation of specific intestinal epithelial progenitors by enteric neurons. Proc Natl Acad Sci U S A.

[53] Farin HF, Karthaus WR, Kujala P, Rakhshandehroo M, Schwank G, Vries RG, Kalkhoven E, Nieuwenhuis EE, Clevers H (2014). Paneth cell extrusion and release of antimicrobial products is directly controlled by immune cell-derived IFN-gamma. J Exp Med.

[54] Sehgal A, Donaldson DS, Pridans C, Sauter KA, Hume DA, Mabbott NA (2018). The role of CSF1R-dependent macrophages in control of the intestinal stem-cell niche. Nat Commun.

[55] Saha S, Aranda E, Hayakawa Y, Bhanja P, Atay S, Brodin NP (2016). Macrophage-derived extracellular vesicle-packaged WNTs rescue intestinal stem cells and enhance survival after radiation injury. Nat Commun.

[56] Ilkka S Junttila. Tuning the cytokine responses: an update on interleukin (IL)-4 and IL-13 receptor complexes. Front Immunol.2018;9:888. 10.3389/fimmu.2018.00888 PMC6001902 29930549

[57] Saito Y, Iwatsuki K, Inaba A, Sato M, Tadaishi M, Shimizu M (2020). Interleukin-4 suppresses the proliferation and alters the gene expression in Enteroids. Cytotechnology..

[58] Pickert G, Neufert C, Leppkes M, Zheng Y, Wittkopf N, Warntjen M (2009). STAT3 links IL-22 signaling in intestinal epithelial cells to mucosal wound healing. J Exp Med.

[59] Camporeale A, Poli V (2012). IL-6, IL-17 and STAT3: a holy trinity in auto-immunity?. Front Biosci.

[60] Lindemans CA, Calafiore M, Mertelsmann AM, O’Connor MH, Dudakov JA, Jenq RR (2015). Interleukin-22 promotes intestinal-stem-cell-mediated epithelial regeneration. Nature.

[61] Glal D, Sudhakar JN, Lu H-H, Liu M-C, Chiang H-Y, Liu Y-C (2018). ATF3 sustains IL-22-induced STAT3 phosphorylation to maintain mucosal immunity through inhibiting phosphatases. Front Immunol.

[62] Jeffery V, Goldson AJ, Dainty JR, Chieppa M, Sobolewski A (2017). Interleukin-6 signaling regulates small intestinal crypt homeostasis. J Immunol.

[63] Mahapatro M, Foersch S, Hefele M, He G-W, Giner-Ventura E, Mchedlidze T (2016). Programming of intestinal epithelial differentiation by IL-33 derived from pericryptal fibroblasts in response to systemic infection. Cell Rep.

[64] Bradford EM, Ryu SH, Singh AP, Lee G, Goretsky T, Sinh P (2017). Epithelial TNF receptor signaling promotes mucosal repair in IBD. J Immunol.

[65] Zhang J (2011). UCP2 regulates energy metabolism and differentiation potential of human pluripotent stem cells. EMBO J.

[66] Wang B, Rong X, Palladino E.N.D, Wang J. Fogelman A. M, Martin M. G, Alrefai W. A, Ford D. A, Tontonoz P, Phospholipid remodeling and cholesterol availability regulate intestinal stemness and tumorigenesis. Cell Stem Cell. 2018; 22: 206–220.e4. 10.1016/j.stem.2017.12.017 PMC5807072 29395055

[67] Peregrina K, Houston M, Farooqui C, Dhima E, Sellers RS, Augenlicht LH (2015). Vitamin D is a determinant of mouse intestinal Lgr5 stem cell functions. Carcinogenesis.

[68] Mihaylova MM, Cheng C-W, Cao AQ, Tripathi S, Mana MD, Bauer-Rowe KE (2018). Fasting activates fatty acid oxidation to enhance intestinal stem cell function during homeostasis and aging. Cell Stem Cell.

[69] Cheng C-W, Biton M, Haber AL, Gunduz N, Eng G, Gaynor LT (2019). Ketone body signaling mediates intestinal stem cell homeostasis and adaptation to diet. Cell.

[70] Chen S, Xia Y, Zhu G, Yan J, Tan C, Deng B, et al. Glutamine supplementation improves intestinal cell proliferation and stem cell differentiation in weanling mice. Food Nutr Res. 2018;62:1439. 10.29219/fnr.v62.1439 PMC6060183 30083086

[71] Laplante M, Sabatini DM (2012). mTOR signaling in growth control and disease. Cell.

[72] Sampson LL, Davis AK, Grogg MW, Zheng Y (2016). mTOR disruption causes intestinal epithelial cell defects and intestinal atrophy Postinjury in mice. FASEB J.

[73] Zhou Y, Rychahou P, Wang Q, Weiss HL, Evers BM (2015). TSC2/mTORC1 signaling controls Paneth and goblet cell differentiation in the intestinal epithelium. Cell Death Dis.

[74] Sengupta S, Peterson TR, Laplante M, Oh S, Sabatini DM (2010). mTORC1 controls fasting-induced ketogenesis and its modulation by ageing. Nature..

[75] Masaki Igarashi , Leonard Guarente mTORC1 and SIRT1 cooperate to foster expansion of gut adult stem cells during calorie restriction. Cell, 2016;166(2):436–450. 10.1016/j.cell.2016.05.044 27345368

[76] Yilmaz ÖH, Katajisto P, Lamming DW (2012). mTORC1 in the Paneth cell niche couples intestinal stem-cell function to calorie intake. Nature..

[77] Schakel K, Kannagi R, Kniep B, Goto Y, Mitsuoka C, Zwirner J (2002). Rieber: 6-Sulfo LacNAc, a novel carbohydrate modification of PSGL-1, defines an inflammatory type of human dendritic cells. Immunity..

[78] Sara H, Rouhanifard ALA, Meng L, Moremen KW, Wu P (2018). Engineered glycocalyx regulates stem cell proliferation in murine crypt organoids. Cell Chem Boil.

[79] Rodríguez-Colman MJ, Schewe M, Meerlo M (2017). Interplay between metabolic identities in the intestinal crypt supports stem cell function. Nature..

[80] Martin K, Kirkwood TB, Potten CS (1998). Age changes in stem cells of murine small intestinal crypts. Exp Cell Res.

[81] Groarke EM, Young NS (2019). Aging and hematopoiesis. Clin Geriatr Med.

[82] Iwamoto T, Ouchi Y (2014). Emerging evidence of insulin-like growth factor 2 as a memory enhancer: a unique animal model of cognitive dysfunction with impaired adult neurogenesis. Rev Neurosci.

[83] Fernandez-Flores A, Saeb-Lima M, Cassarino DS (2019). Histopathology of aging of the hair follicle. J Cutan Pathol.

[84] Barroso-Vilares M, Logarinho E (2019). Chromosomal instability and pro-inflammatory response in aging. Mech Ageing Dev.

[85] Papadopoli D, Boulay K, Kazak L, Pollak M, Mallette F, Topisirovic I, Hulea L (2019). mTOR as a central regulator of lifespan and aging. F1000Res.

[86] Moorefield EC, Andres SF, Blue RE, Van Landeghem L, Mah AT (2017). Aging effects on intestinal homeostasis associated with expansion and dysfunction of intestinal epithelial stem cells. Aging (Albany).

[87] He D, Wu H, Xiang J, Ruan X, Peng P, Ruan Y (2020). Gut stem cell aging is driven by mTORC1 via a p38 MAPK-p53 pathway. Nat Commun.

[88] Nalapareddy K, Nattamai KJ, Kumar RS (2017). Canonical Wnt signaling ameliorates aging of intestinal stem cells. Cell Rep.

[89] Martin K, Potten CS, Roberts SA, Kirkwood TB (1998). Altered stem cell regeneration in irradiated intestinal crypts of senescent mice. J Cell Sci.

[90] Moorefield EC, Andres SF, Blue RE (2017). Aging effects on intestinal homeostasis associated with expansion and dysfunction of intestinal epithelial stem cells. Aging (Albany NY).

[91] Pentinmikko N, Iqbal S, Mana M (2019). Notum produced by Paneth cells attenuates regeneration of aged intestinal epithelium. Nature.

[92] Kakugawa S (2015). Notum deacylates Wnt proteins to suppress signaling activity. Nature..

[93] Igarashi M, Miura M, Williams E, Jaksch F, Kadowaki T, Yamauchi T (2019). NAD+ supplementation rejuvenates aged gut adult stem cells. Aging Cell.

[94] Calvert R, Pothier P (1990). Migration of fetal intestinal intervillous cells in neonatal mice. Anat Rec.

[95] Moxey PC, Trier JS (1978). Specialized cell types in the human fetal small intestine. Anat Rec.

[96] Mallow EB, Harris A, Salzman N, Russell JP, DeBerardinis RJ, Ruchelli E (1996). Human enteric defensins. Gene structure and developmental expression. J Biol Chem.

[97] van Es JH, van Gijn ME, Riccio O, van den Born M, Vooijs M, Begthel H (2005). Notch/gamma-secretase inhibition turns proliferative cells in intestinal crypts and adenomas into goblet cells. Nature..

[98] van Es JH (2012). A critical role for the Wnt effector Tcf4 in adult intestinal homeostatic self-renewal. Mol Cell Biol.

[99] Heuberger J, Kosel F, Qi J, Grossmann KS (2014). Shp2/MAPK signaling controls goblet/paneth cell fate decisions in the intestine. Proc Natl Acad Sci U S A.

[100] Bänziger C, Soldini D, Schütt C, Zipperlen P, Hausmann G, Basler K (2006). Wntless, a conserved membrane protein dedicated to the secretion of Wnt proteins from signaling cells. Cell.

[101] Bartscherer K, Pelte N, Ingelfinger D, Boutros M (2006). Secretion of Wnt ligands requires Evi, a conserved transmembrane protein. Cell..

[102] Das S, Yu S, Sakamori, Vedula P (2015). Rab8a vesicles regulate Wnt ligand delivery and Paneth cell maturation at the intestinal stem cell niche. Development..

[103] Jones JT, Akita RW, Sliwkowski MX (1999). Binding specificities and affinities of egf domains for ErbB receptors. FEBS Lett.

[104] Almohazey D, Lo YH, Vossler CV (2017). The ErbB3 receptor tyrosine kinase negatively regulates Paneth cells by PI3K-dependent suppression of Atoh1. Cell Death Differ.

[105] Pin CL, Bonvissuto AC, Konieczny SF (2000). Mist1 expression is a common link among serous exocrine cells exhibiting regulated exocytosis. Anat Rec.

[106] Dekaney CM, King S, Sheahan B, Cortes JE (2019). Mist1 expression is required for Paneth cell maturation. Cell Mol Gastroenterol Hepatol.

[107] Watanabe N, Mashima H, Miura K, Goto T, Yoshida M, Goto A (2016). Requirement of Gαq/Gα11 signaling in the preservation of mouse intestinal epithelial homeostasis. Cell Mol Gastroenterol Hepatol.

[108] Pellegrinet L, Rodilla V, Liu Z (2011). Dll1- and dll4-mediated notch signaling are required for homeostasis of intestinal stem cells. Gastroenterology.

[109] Moscat J, Diaz-Meco MT, Wooten MW (2009). Of the atypical PKCs, Par-4 and p62: recent understandings of the biology and pathology of a PB1-dominated complex. Cell Death Differ.

[110] Riccio O, van Gijn ME, Bezdek AC, Pellegrinet L, van Es JH, Zimber-Strobl U, Strobl LJ, Honjo T, Clevers H, Radtke F (2008). Loss of intestinal crypt progenitor cells owing to inactivation of both Notch1 and Notch2 is accompanied by derepression of CDK inhibitors p27Kip1 and p57Kip2. EMBO Rep.

[111] Yin X, Farin HF, van Es JH, Clevers H, Langer R, Karp JM (2014). Niche independent high-purity cultures of Lgr5+ intestinal stem cells and their progeny. Nat Methods.

[112] Nakanishi Y, Reina-Campos M, Nakanishi N (2016). Control of Paneth cell fate, intestinal inflammation, and tumorigenesis by PKCλ/ι. Cell Rep.

[113] Jeon MK, Kaemmerer E, Schneider U, Schiffer M, Klaus C, Hennings J (2018). Notch inhibition counteracts Paneth cell death in absence of caspase-8. Virchows Arch.

[114] Gebert N, Cheng C-W, Kirkpatrick JM, Di Fraia D, Yun J, Schädel P (2020). Region-specific proteome changes of the intestinal epithelium during aging and dietary restriction. Cell Rep.

[115] Gao Y, Yan Y, Tripathi S, Pentinmikko N, Amaral A, Päivinen P (2020). LKB1 represses ATOH1 via PDK4 and energy metabolism and regulates intestinal stem cell fate. Gastroenterology.

[116] Dakota N Jackson, Marina Panopoulos, William L Neumann, Kevin Turner, Brandi L Cantarel, LuAnn Thompson-Snipes et al. Mitochondrial dysfunction during loss of prohibitin 1 triggers Paneth cell defects and ileitis. Gut. 2020 gutjnl-2019-319523. [Epub ahead of print]. 10.1136/gutjnl-2019-319523 PMC7483170 32111635

[117] Sevana Khaloian , Eva Rath , Nassim Hammoudi , Elisabeth Gleisinger , Andreas Blutke , Pieter Giesbertz . Mitochondrial impairment drives intestinal stem cell transition into dysfunctional Paneth cells predicting Crohn’s disease recurrence. Gut. 2020 gutjnl-2019-319514. [Epub ahead of print]. 10.1136/gutjnl-2019-319514 PMC7569388 32111634

[118] Han SJ, Li H, Kim M, Vivette D'A, Thomas Lee H (2019). Intestinal toll-like receptor deficiency leads to Paneth cell hyperplasia and exacerbates kidney, intestine, and liver injury after ischemia/reperfusion injury. Kidney Int.

[119] Wang J, Tian F, Zheng H, Tian H, Wang P, Zhang L (2017). N-3 polyunsaturated fatty acid-enriched lipid emulsion improves Paneth cell function via the IL-22/Stat3 pathway in a mouse model of total parenteral nutrition. Biochem Biophys Res Commun.

[120] Liu R, Moriggl R, Zhang D, Li H, Karns R, Ruan H-B (2019). Constitutive STAT5 activation regulates Paneth and Paneth-like cells to control Clostridium difficile colitis. Life Sci Alliance.

[121] Günther C, Ruder B, Stolzer I (2019). Interferon lambda promotes paneth cell death via STAT1 signaling in mice and is increased in inflamed ileal tissues of patients with Crohn’s disease. Gastroenterology.

[122] Mikuda N, Schmidt-Ullrich R, Kärgel E, Golusda L, Wolf J, Höpken UE, Scheidereit C (2020). Deficiency in IκBα in the intestinal epithelium leads to spontaneous inflammation and mediates apoptosis in the gut. J Pathol.

[123] Alvarado DM, Chen B, Iticovici M, Thaker AI, Dai N, VanDussen KL (2019). Epithelial indoleamine 2,3-dioxygenase 1 modulates aryl hydrocarbon receptor and notch signaling to increase differentiation of secretory cells and alter mucus-associated microbiota. Gastroenterology.

[124] Ayabe T, Satchell DP, Wilson CL, Parks WC, Selsted ME, Ouellette AJ (2000). Secretion of microbicidal alpha-defensins by intestinal Paneth cells in response to bacteria. Nat Immunol.

[125] Bar SE, Friedman A (2018). Innate immune functions of avian intestinal epithelial cells: response to bacterial stimuli and localization of responding cells in the developing avian digestive tract. PLoS One.

[126] Cobo ER, Holani R, Moreau F, Nakamura K, Ayabe T, Mastroianni JR, Ouellette A, Chadee K (2018). Entamoeba histolytica alters ileal Paneth cell functions in intact and Muc2 mucin deficiency. Infect Immun.

